# 4,4′-Diaponeurosporene from *Lactobacillus plantarum* subsp. *plantarum* KCCP11226: Low Temperature Stress-Induced Production Enhancement and In Vitro Antioxidant Activity

**DOI:** 10.4014/jmb.2010.10022

**Published:** 2020-11-04

**Authors:** Mibang Kim, Dong-Hyun Jung, Dong-Ho Seo, Young-Seo Park, Myung-Ji Seo

**Affiliations:** 1 Department of Bioengineering and Nano-Bioengineering, Graduate School of Incheon National University, Incheon 2202, Republic of Korea; 2 Bacteria Research Team, Nakdonggang National Institute of Biological Resources, Sangju 374, Republic of Korea; 3 Department of Food Science and Technology, College of Agriculture and Life Sciences, Jeonbuk National University, Jeonju 54896, Republic of Korea; 4 Department of Agricultural Convergence Technology, Jeonbuk National University, Jeonju 5896, Republic of Korea; 5 Department of Food Science and Biotechnology, Gachon University, Seongnam 13120, Republic of Korea; 6 Division of Bioengineering, Incheon National University, Incheon 22012, Republic of Korea; 7 Institute for New Drug Development, Incheon National University, Incheon 22012, Republic of Korea

**Keywords:** *Lactobacillus plantarum* subsp. *plantarum*, 4,4'-diaponeurosporene, C_30_ carotenoid, antioxidant, low temperature

## Abstract

Carotenoids, which have biologically beneficial effects and occur naturally in microorganisms and plants, are pigments widely applied in the food, cosmetics and pharmaceutical industries. The compound 4,4'-diaponeurosporene is a C_30_ carotenoid produced by some *Lactobacillus* species, and *Lactobacillus plantarum* is the main species producing it. In this study, the antioxidant activity of 4,4'-diaponeurosporene extracted from *L. plantarum* subsp. *plantarum* KCCP11226 was examined. Maximum carotenoid content (0.74 ± 0.2 at A_470_) was obtained at a relatively low temperature (20°C). The DPPH radical scavenging ability of 4,4'-diaponeurosporene (1 mM) was approximately 1.7-fold higher than that of butylated hydroxytoluene (BHT), a well-known antioxidant food additive. In addition, the ABTS radical scavenging ability was shown to be 2.3- to 7.5-fold higher than that of BHT at the range of concentration from 0.25 mM to 1 mM. The FRAP analysis confirmed that 4,4'- diaponeurosporene (0.25 mM) was able to reduce Fe^3+^ by 8.0-fold higher than that of BHT. Meanwhile, 4,4'-diaponeurosporene has been confirmed to be highly resistant to various external stresses (acid/bile, high temperature, and lysozyme conditions). In conclusion, *L. plantarum* subsp. *plantarum* KCCP11226, which produces 4,4'-diaponeurosporene as a functional antioxidant, may be a potentially useful strain for the development of functional probiotic industries.

## Introduction

Carotenoids, natural pigments belonging to the isoprenoid group, exhibit yellow, orange, red, and purple colors and naturally occur in algae, yeasts, fungi, bacteria, and plants [1, 2]. Carotenoids have various functions that are beneficial to human health; hence, they have been widely applied in the food, cosmetic, and pharmaceutical industries [1, 2]. Representatively, they have antioxidant, anticancer, and antimicrobial activity, as well as immune regulatory functions [2].

Recently, scientific interest related to carotenoid production using microorganisms has been increasing. Microbial pigments are a better alternative to synthetic or plant pigments because of their safety, availability, non-seasonality, scalability, high yield, and reduced process [2]. Most carotenoids found in bacteria consist of eight isoprene units with a 40-carbon structure (tetraterpenoids, C_40_ carotenoids) such as astaxanthin, lycopene, and β-carotene. However, triterpenoids (C_30_ carotenoids) have been reported to be found only in several bacterial species, such as *Lactobacillus plantarum*, *Enterococcus faecium*, *Staphylococcus aureus*, *Methylobacterium rhodinum*, and some heliobacteria [3].

*L. plantarum* is a widespread member of the genus of *Lactobacillus*, commonly found in ecological niches such as various dairy products, vegetables, fish and gastrointestinal tracts [4]. It has been extensively used as a probiotic strain to provide various beneficial effects on human health [5]. Recently, several biotechnological studies have reported on *L. plantarum*, which can produce the C_30_ carotenoid 4,4′-diaponeurosporene. In general, bacterial carotenoids can be produced from farnesyl pyrophosphate (FPP), a product of the mevalonate pathway [6]. Garrido-Fernández *et al*. (2010) reported that 4,4′-diaponeurosporene can be synthesized through a series of FPP desaturation steps by dehydrosqualene synthase (*crtM*) and dehydrosqualene desaturase (*crtN*) in *L. plantarum* [3]. In addition, Turpin *et al*. (2016) suggested that the identification of *crtM*-*crtN* genes, accompanied by biochemical analysis, is an efficient method for screening C_30_ carotenoid-producing lactic acid bacteria [7]. Our previous studies reported that *L. plantarum* subsp. *plantarum* KCCP11226 isolated from kimchi, a Korean fermented vegetable, induced 4,4′-diaponeurosporene production under various stress environments by upregulating the carotenoid biosynthetic operon *crtM*-*crtN* [8, 9]. Furthermore, they showed that the *crtM*–*crtN* operon can be detected in some *Lactobacillus* species, including *L. paraplantarum*, *L. paracasei*, *L. herbarum*, *L. mundanjiangensis*, and *L. florum*, but it is found in most *L. plantarum* species, and is well conserved [9].

One of the important properties of carotenoids is to act as antioxidants that can protect cells and tissues from reactive oxygen species (ROS) damage [10, 11]. In general, the harmful effects of ROS on cells are most often cell death due to damage to cell structures, including lipids and membranes, proteins, and nucleic acids [12]. Indeed, most of the focus on carotenoids in recent years has been to understand their function, especially as antioxidants [13]. Recently, the resistance of 4,4′-diaponeurosporene to oxidative stress has been reported [8, 14]. This carotenoid is considered to act as an antioxidant by removing free radicals through conjugated double bonds [3, 15, 16]. As described above, *L. plantarum*, a main species of *Lactobacillus* that produces 4,4′-diaponeurosporene with the *crtM*–*crtN* operon, is important. Although the antioxidant activities of various types of carotenoids have been studied, that of 4,4′-diaponeurosporene from *L. plantarum* is still unclear.

In this study, we evaluated the antioxidant activity of 4,4′-diaponeurosporene produced from *L. plantarum* subsp. *plantarum* KCCP11226 through several kinds of antioxidant measurements. In addition, the availability of strain KCCP11226 for probiotic use was investigated through in vitro experiments on safety and functionalities such as antibiotic resistance, cell surface hydrophobicity, and stress tolerance, including acid/bile, high temperature, and lysozyme. To the best of our knowledge, this study is the first report on the antioxidant activity of 4,4′-diaponeurosporene produced by *Lactobacillus*.

## Materials and Methods

### Bacterial Strain and Reagents

*L. plantarum* subsp. *plantarum* KCCP11226 was previously isolated from a Korean fermented food [8]. *L. rhamnosus* GG (KCTC 5033), which was used as reference strain in this study, was purchased from the Korean Collection for Type Cultures (KCTC, Korea). De Man Rogosa and Sharpe (MRS) broth was purchased from MBcell (Korea) and oxgall was obtained from Difco Laboratories (USA). Antibiotic agents including ampicillin, erythromycin, gentamicin, ciprofloxacin, lincomycin, novobiocin, tetracycline, and streptomycin were purchased from Sigma-Aldrich (USA). All other chemicals used in this study were of analytical grade and obtained from Sigma-Aldrich.

### Cell Surface Hydrophobicity

Bacterial cell surface hydrophobicity was evaluated according to Ekmekci *et al*. (2009), with slight modifications [17]. Briefly, KCCP11226 was cultivated at 30°C for 24 h and then harvested by centrifugation at 10,000 ×*g* for 10 min, followed by washing twice with PBS (pH 7.0). The pellet was resuspended in PBS buffer to an optical density (OD) of 0.6. One milliliter of test hydrocarbons (xylene or toluene) was added to the cell suspension and mixed for 2 min. The two phases were separated by incubation for 30 min at room temperature. Afterward, the aqueous phases were measured by absorbance at 600nm. The percentage of cell surface hydrophobicity was calculated using the following equation: Hydrophobicity (%) = [(A_0_ – A_1_)/A_0_] × 100, where A_0_ and A_1_ represent the absorbance at 600 nm before and after mixing, respectively.

### Antibiotic Resistance

KCCP11226 was cultivated at 30°C for 24 h (100 μl) and then spread on MRS agar medium, and antibiotic discs containing ampicillin (100 μg/ml), erythromycin (25 μg/ml), gentamicin (30 μg/ml), ciprofloxacin (10 μg/ml), lincomycin (15 μg/ml), novobiocin (10 μg/ml), tetracycline (30 μg/ml), and streptomycin (50 μg/ml) were placed on the inoculated plates. All plates were incubated at 30°C for 24 h and the inhibition zones were measured.

### Survival Under Environmental Stress

Survival rates after exposure to stress were evaluated according to Hagi *et al*. (2013) [14]. KCCP11226 was cultured in MRS medium for 24 h at 30°C, harvested by centrifugation at 8,000 ×*g* for 10 min, and rinsed twice with saline. The rinsed bacterial pellets were resuspended in 500 μl of low-pH saline (pH 1.5 or 2.0, adjusted with HCl), oxgall (10% or 20%), or lysozyme (8 or 12mg/ml) to determine survival rate under each of the environmental stresses. Under each of these stress conditions, the bacterial suspension was exposed to 30°C for 90 min (acid and bile conditions) or 180 min (lysozyme conditions). After that, cells were harvested and washed twice with saline, followed by resuspension in saline (500 μl). Meanwhile, to investigate heat tolerance, the rinsed bacterial pellets resuspended in 500 μl of saline were exposed at 55°C or 60°C for 20 min. The number of cell colonies before and after exposure to heat was counted by viable cells on MRS agar. The viable cells were counted and calculated using the following equation:

Survival rate (%) = (Number of cells after exposure to conditions/initial number of cells) ×100

### Temperature Stress-Induced Carotenoid Production

To create a seed culture, strain KCCP11226 was statically incubated at 30°C for 18 h in MRS broth. Seed culture was inoculated (1%, v/v) into MRS medium (3 L) in an Erlenmeyer flask (5 L) and the flask was statically incubated at 30°C. Samples (100 ml) were taken every 2 h. Next, to study the effect of temperature on carotenoid production, additional cultures were incubated, changing only the temperature, under the same culture conditions. The bacterial cultures incubated at 10°C, 20°C, 30°C, and 40°C were taken after 24 h incubation. The cell density (OD_600_) and crude carotenoid (A_470_) of the collected samples were measured.

### Extraction and Purification of Carotenoid

The yellow pigments of strain KCCP11226 were extracted according to our previous study [9]. In brief, 100 ml of cultured cells were harvested by centrifugation at 10,000 ×*g* for 10 min and extracted in 5 ml methanol overnight. Next, 5ml of hexane and 2.5ml of distilled water were added to the methanol extract. After centrifugation at 8,000 ×*g* for 10 min, the organic phase containing carotenoids was transferred to a 15 ml tube. The organic phase was evaporated and then dissolved in 1 ml of petroleum ether. The pigmentation levels of crude carotenoids were measured at an absorption wavelength of 470 nm (A_470_) using a spectrophotometer (Shimadzu, Japan).

For purification of 4,4′-diaponeurosporene, the isolated carotenoids were salted out with 5 N NaCl solution and re-extracted with an equal volume of ethyl acetate (EtOAc). The upper phase was loaded onto an anhydrous sodium sulfate column (BioBasic, Canada) for dehydration and then evaporated. The dried carotenoids were dissolved in EtOAc, filtered, and purified by loading on a silica gel column [18]. The total amount of purified carotenoids was quantified using the previously reported extinction coefficient of 4,4′-diaponeurosporene [19, 20].

### Antioxidant Activity of 4,4′-Diaponeurosporene

The scavenging activity of 2,2-diphenyl-1-picrylhydrazyl (DPPH) free radical was determined according to the method described by Kim *et al*. (2019) with slight modifications [8]. The 4,4′-diaponeurosporene was dissolved in DMSO at final concentrations of 0.25, 0.5, and 1 mM, and 100 μl of each solution was added to equal volumes of ethanol containing 0.2 mM DPPH. The mixture was left for 30 min at room temperature, and then the absorbance of the resulting solution was measured at 517 nm (A_517_). The scavenging ability was expressed as follows: scavenging activity (%) = (1 – [A_sample_ – A_blank_]/A_control_) × 100. As a control, the same reactant that did not contain 4,4'-diaponeurosporene was used. Butylated hydroxytoluene (BHT) was also assayed as an antioxidant reference.

The 4,4′-diaponeurosporene was further characterized by the 2,2′-azino-bis(3-ethylbenzothiazoline-6-sulfonic acid) (ABTS) cation radical assay with a slight modification of the method described by Yang *et al*. (2017)[21]. First, ABTS radical solution was generated after reaction of 7 mM ABTS in 20 mM sodium acetate buffer (pH 4.5) with an oxidant (2.45 mM potassium persulfate) in the dark for 12–16 h at 4°C before use. The ABTS radical solution was diluted with 20 mM sodium acetate buffer to an absorbance of 0.7 ± 0.02 at 734 nm. After adding 100 μl of 4,4′-diaponeurosporene dissolved in DMSO to an equal volume of ABTS radical solution, the absorbance was measured at 734 nm (A734) after incubation for 10 min in the dark. The ABTS radical scavenging activity of 4,4'-diaponeurosporene was expressed as Trolox equivalent antioxidant capacity.

The ferric reducing antioxidant power (FRAP) of 4,4′-diaponeurosporene was estimated according to Han *et al*.(2017) [22]. Briefly, FRAP reagents were prepared by mixing acetate buffer (300 mM, pH 3.6), 10 mM 2,4,6-Tris(2-pyridyl)-s-triazine (TPTZ) solution in 40 mM HCl, and 20 mM FeCl_3_·6H_2_O at 10:1:1 (v/v/v). The FRAP reagent solution was maintained at 37°C. Then, 100 μl of 4,4′-diaponeurosporene was allowed to react with 3 ml of FRAP reagent solution and 300 μl of ultrapure water for 5 min at room temperature in the dark. The absorbance values of the mixture were measured at 593 nm (A_593_). The FRAP values were expressed as FeSO_4_ (mM) equivalents using the FeSO_4_·7H_2_O standard curve.

### Statistical Analysis

The results of the experiments were expressed as means ± standard deviations of three independent measurements. The significance of differences in means was evaluated using analysis of variance (ANOVA) with Tukey-Kramer multiple comparison tests, unless mentioned otherwise. The FRAP data was analyzed using an unpaired *t*-test with Welch’s correction. Differences with *p* values of less than 0.01 were considered significant.

## Results and Discussion

### Probiotic Properties of *L. plantarum* subsp. *plantarum* KCCP11226

To produce effective probiotic strains, the safety and functionalities such as antibiotic resistance, adhesion to intestinal mucosa, and stress tolerance (*i.e.*, acid/bile) should be investigated using reliable in vitro/vivo experiments [23]. Accordingly, the antibiotic resistance of *L. plantarum* subsp. *plantarum* KCCP11226 was tested. As a result, it was confirmed to have resistance to erythromycin, gentamicin, ciprofloxacin, lincomycin, tetracycline, and streptomycin, but not against ampicillin and novobiocin (Table S1). The ability to adhere to the intestinal mucosa is a prerequisite for bacterial colonization and is one of the most important properties for ideal probiotic strains. Adherence of bacterial cells is usually correlated with cell surface hydrophobicity [24]. The cell surface hydrophobicity of strain KCCP11226 producing 4,4′-diaponeurosporene was measured and showed 66.1± 2.2% and 96.2 ± 0.5% against xylene and toluene, respectively (Fig. S1). These values were significantly higher than those of *L. rhamnosus* GG as the most well-studied probiotic bacterium, which showed 56.7% and 82.8%hydrophobicity against xylene and toluene, respectively (*p* < 0.01).

**Fig. 1:**
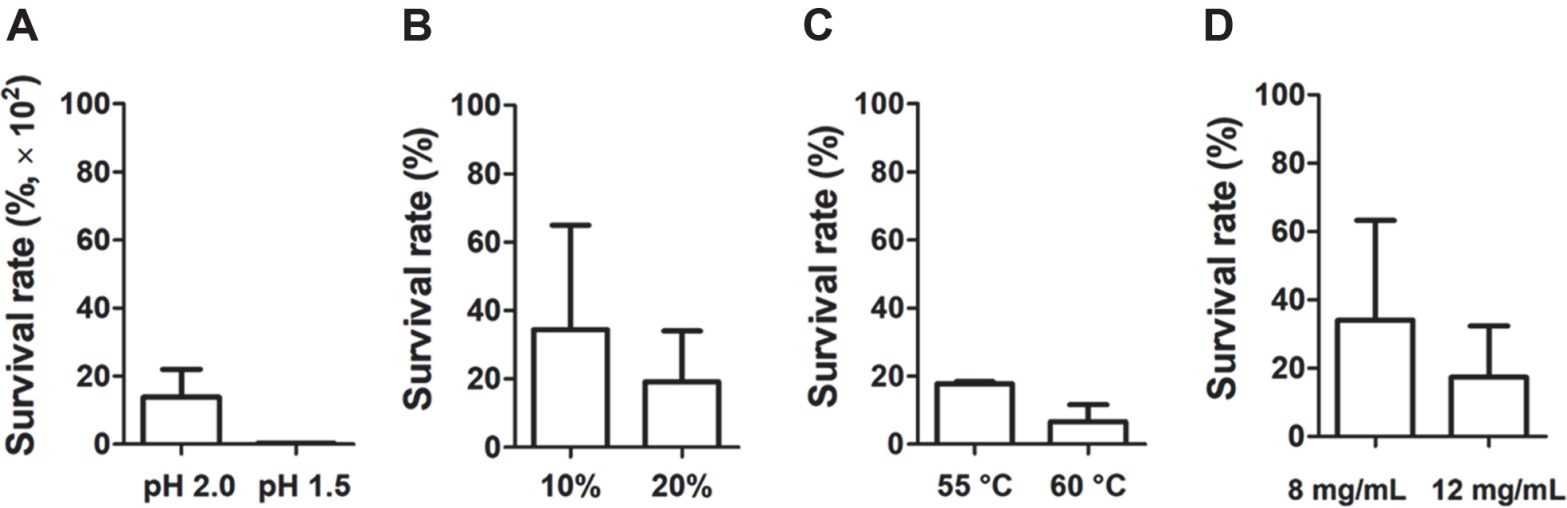
Survival rate of *L. plantarum* subsp. *plantarum* KCCP11226 after exposure to external stress. (**A**) Low pH, (**B**) bile acid, (**C**) high temperatures, (**D**) lysozyme. The bacterial cells were exposed to 30°C for 90 min (acid and bile conditions), 20 min (high temperature conditions), and 180 min (lysozyme conditions).

In addition, the ability to survive after ingestion is an important characteristic of probiotic microorganisms because they are exposed to low pH and bile acids during passage through the digestive tract. As shown in Fig. 1A, strain KCCP11226, when exposed to fairly severe acid (pH 2.0 and 1.5) for 90 min, showed survival rates of 0.139%and 0.003%, respectively. However, considering the initial number of cells (7.3 × 10^7^ and 6.0×10^7^ CFU/ml, respectively), we confirmed that a significant number of cells still survived (1.0×10^5^ and 1.8×10^3^ CFU/ml, respectively). The resistance of strain KCCP11226 to bile acid was tested at 10% and 20% oxgall. Considering that a typical bile acid resistance experiment was performed at less than 1% oxgall [25], the applied concentrations were very harsh conditions. However, interestingly, we confirmed that over 34.5 ± 30.4% and 19.1 ± 15.0% of cells can survive under the treatments of 10% and 20% oxgall, respectively (Fig. 1B). In addition, heat stress during food manufacturing and lysozyme in dairy products are important factors influencing cell death [14]; hence, the resistance of strain KCCP11226 to them was investigated. The survival rates against heat stress were 17.8 ± 0.6%and 6.7 ± 4.9% at 55°C and 60°C, respectively (Fig. 1C). Furthermore, 34.0 ± 29.2% and 17.4 ± 15.0% of the initial cells survived at 8 and 12 mg/mL of lysozyme exposure, respectively (Fig. 1D). In addition to the above-mentioned external stresses, our previous study also revealed that the strain KCCP11226 showed the high survival rates (0.79% and 0.11%) under oxidative stress after exposure to H_2_O_2_ at concentrations of 16 mM and 32 mM, respectively [8].

Taken together, these results indicate that *L. plantarum* subsp. *plantarum* KCCP11226 has considerable potential as a probiotic strain because it shows survival under very harsh acid and bile conditions as well as external stress such as heat and lysozyme.

### Carotenoid Production of *L. plantarum* subsp. *plantarum* KCCP11226 Induced by Low Temperature Stress

**Fig. 2:**
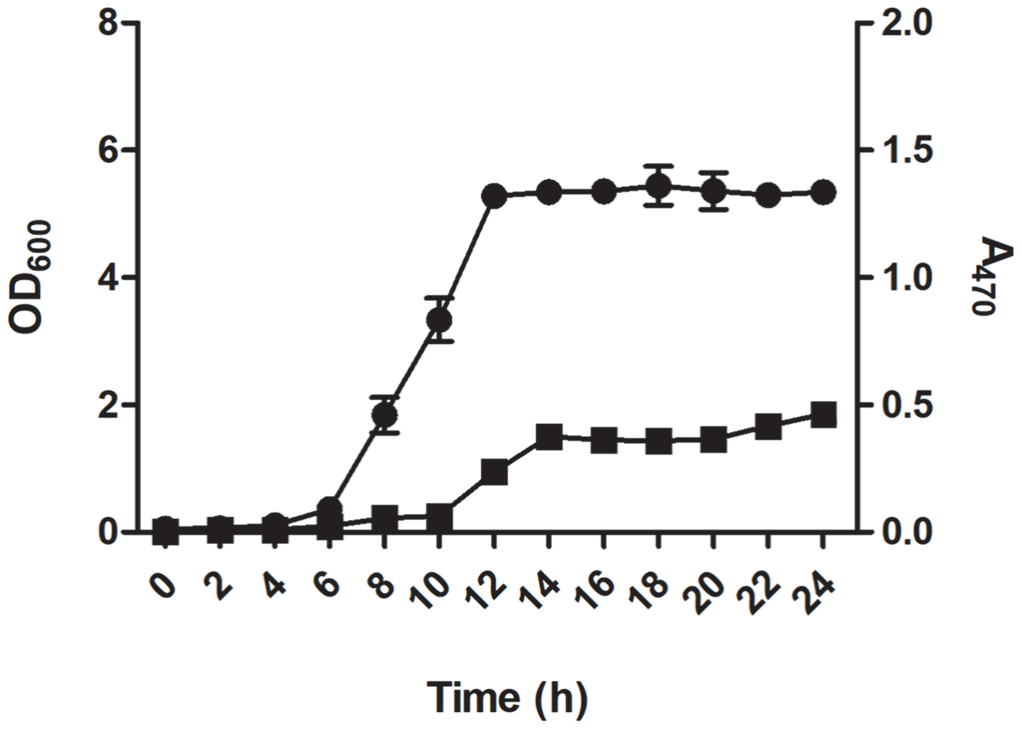
Time profiles of cell growth and carotenoid production of *L. plantarum* subsp. *plantarum* KCCP11226. The bacterial cells were statically grown at 30°C for 24 h. Circles and squares indicate cell growth (600 nm) and carotenoid production (470 nm), respectively.

The time profiles of cell growth and C_30_ carotenoid production by strain KCCP11226 were investigated under static growth conditions at 30°C. The cell growth and carotenoid levels were measured every 2 h. After 12 h of incubation, cell growth reached the stationary phase and the time profile of C_30_ carotenoid production exhibited with similar trends to that of cell growth, showing the maximal carotenoid production of 0.46 (A_470_) after 24 h of cultivation (Fig. 2).

**Fig. 3:**
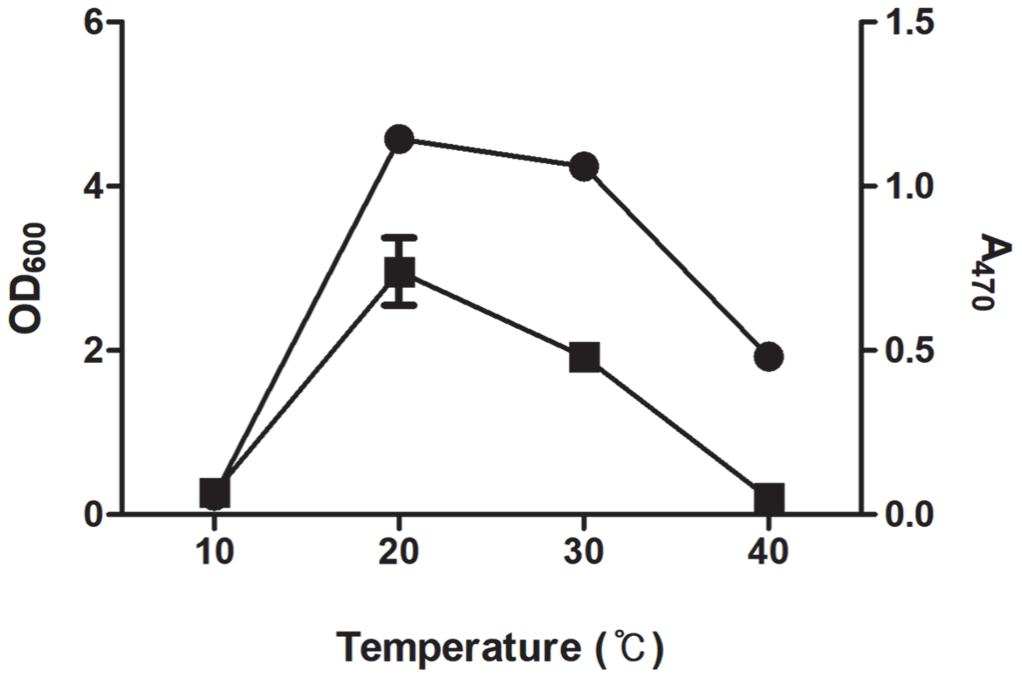
Effect of cultivation temperature on cell growth and carotenoid production of *L. plantarum* subsp. *plantarum* KCCP11226. The bacterial cells were statically grown at various temperatures (10°C, 20°C, 30°C, and 40°C) for 24 h. Circles and squares indicate cell growth (600 nm) and carotenoid production (470 nm), respectively.

The induction of carotenoid production by strain KCCP11226 was investigated through 24 h culture at various temperatures (10°C, 20°C, 30°C, and 40°C) (Fig. 3). As a result, the cells hardly grew at the low temperature of 10°C; hence, no carotenoid was produced. In addition, the cells grew in significant amounts up to 1.93 (OD_600_) at a relatively high temperature of 40°C, but little carotenoid (0.05 at A_470_) was produced. On the other hand, at relatively mild temperatures of 20°C and 30°C, the cells grew at 4.58 and 4.24 (OD_600_), respectively. However, there was a significant difference in carotenoid production. At 30°C, 0.48 (A_470_) of carotenoid was produced, but a much higher amount of 0.74 ± 0.2 (A_470_) was produced at 20°C, which is a relatively low temperature. Temperature is a significant factor affecting the carotenoid biosynthetic pathway depending on the specificity of microbial species, followed by the quantity variation of produced carotenoids [26]. Simpson *et al*. (1964) investigated the increase of β-carotene production from oleaginous red yeast *Rhodotorula glutinis* by decrease of culture temperature [27]. Another previous study also reported that the representative carotenoid produced by red yeast *Phaffia rhodozyma* was changed from torularhodin to astaxanthin by decrease of culture temperature from 30°C to 20°C [28].

In fact, our previous study already investigated the enhancement of C_30_ carotenoid production from *L. plantarum* subsp. *plantarum* KCCP11226 by employing the oxidative and salt stresses, which resulted in aerobic condition and high concentration of sodium chloride having positive effects on the increase of carotenoid production [9]. Likewise, various factors affecting carotenogenesis in microorganisms, such as light, metal ions, and chemical agents, have also been reported [29]. However, most of these studies have been limited to yeast producing C_40_ carotenoids [26], while carotenogenesis studies targeting lactic acid bacteria have not been actively conducted. Therefore, further studies are needed on the strategies for improving the production yield of microbial carotenoid, in particular carotenoid-biosynthesizing lactic acid bacteria as alternative sources of natural C_30_ carotenoids.

### Antioxidant Activity of 4,4'-Diaponeurosporene Extracted from *L. plantarum* subsp. *plantarum* KCCP11226

**Fig. 4:**
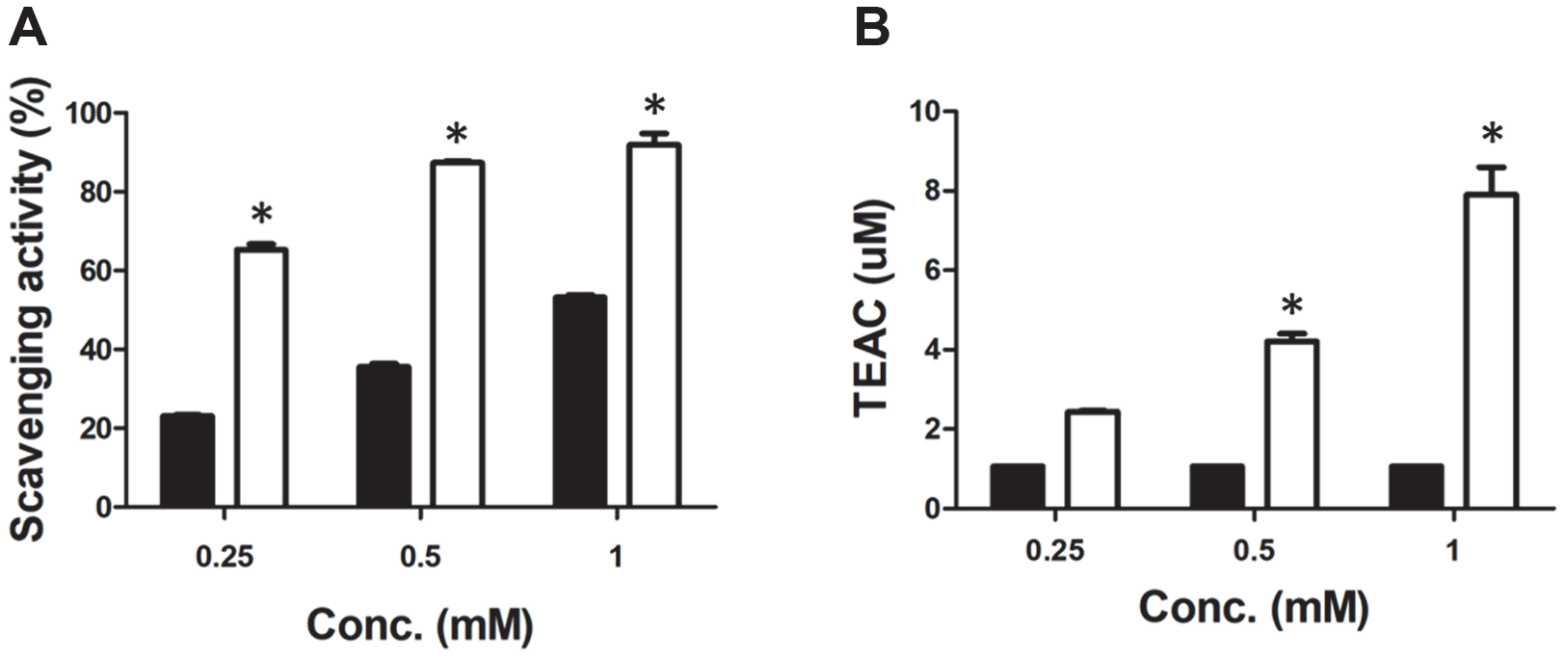
Antioxidant activity of 4,4'-diaponeurosporene extracted from *L. plantarum* subsp. *plantarum* KCCP11226. (**A**) DPPH and (**B**) ABTS scavenging activity of the 4,4′-diaponeurosporene. Black and white bars show BHT and 4,4'-diaponeurosporene, respectively. The results from three independent tests are represented as means ± SD. Significant means were expressed after analysis of variance (ANOVA) with Tukey-Kramer multiple comparison tests (*p < 0.01).

Radical scavenging assays using DPPH and ABTS radicals are widely used to evaluate the antioxidant activity of biological samples [30]. In this study, 4,4′-diaponeurosporene extracted from strain KCCP11226 showed significantly higher DPPH free radical scavenging activity than the same concentration of butylated hydroxytoluene (BHT), which is a well-known antioxidant food additive (Fig. 4A). At 0.25, 0.5, and 1 mM, 4,4′-diaponeurosporene had 65.3 ± 2.5%, 87.4 ± 0.7%, and 92.0 ± 4.9% DPPH radical scavenging activity, respectively, whereas BHT showed 23.1 ± 0.4%, 35.7 ± 1.3%, and 53.2 ± 1.0%, respectively. In previous studies, fucoxanthin showed 70% DPPH radical scavenging activity at a concentration of 300 mM [11]. In the case of astaxanthin and β-carotene, DPPH radical scavenging activity was shown to be 52% and 36% at 1.5 mM concentration, respectively [31]. As a result, we confirmed that 4,4′-diaponeurosporene had significantly better DPPH radical scavenging activity than other carotenoids.

**Fig. 5:**
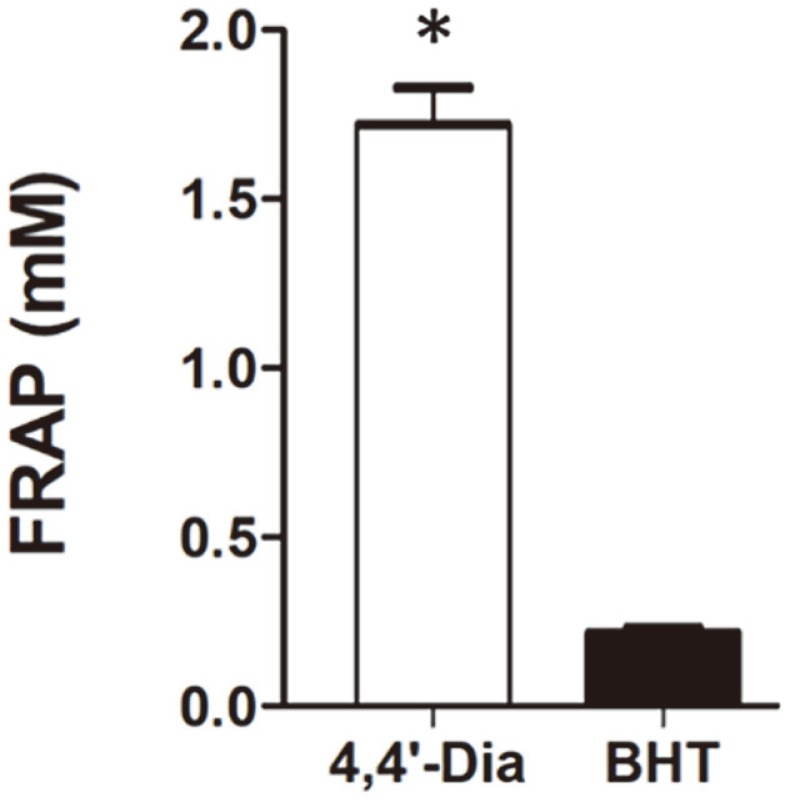
FRAP values of 4,4'-diaponeurosporene extracted from *L. plantarum* subsp. *plantarum* KCCP11226. The applied concentrations are 0.25 mM each of 4,4′-diaponeurosporene (4,4′-Dia) and BHT. The results from three independent tests are represented as means ± SD. Significant means were calculated by the unpaired t-test with Welch’s correction (*p < 0.01).

In addition, 4,4′-diaponeurosporene effectively scavenged ABTS radicals in a concentration-dependent manner. In the Trolox equivalent antioxidant capacity (TEAC) analysis using Trolox as a reference compound, 0.25, 0.5, and 1 mM of 4,4′-diaponeurosporene showed 2.4 ± 0.1, 4.2 ± 0.3, and 7.9 ± 1.2 μM of TEAC value, respectively (Fig. 4B). In contrast, only 1.1 μM of TEAC value was observed in all cases of the corresponding BHT concentration. Moreover, the FRAP assay was also performed because it is closely related to other antioxidant properties as an indicator of electron donating activity [32]. The effect of antioxidants on ferric reducing activity is primarily due to the size of the conjugated double bond system [33]. As a result, 4,4′-diaponeurosporene showed FRAP values of 1.7 ± 0.1, which was 8.0-fold higher compared to that of BHT (0.2) at the same concentration (0.25 mM) (Fig. 5).

In conclusion, *L. plantarum* subsp. *plantarum* KCCP11226, which is the main *Lactobacillus* species producing, 4,4′-diaponeurosporene, satisfies the conditions for use as a probiotic strain by surviving under various harsh conditions. In addition, 4,4′-diaponeurosporene, a C_30_ carotenoid produced by strain KCCP11226, exhibits good antioxidant activity. Therefore, strain KCCP11226 may be a useful functional antioxidant producer and a strain that can also be applied in the probiotic industry.

## Supplemental Material

Supplementary data for this paper are available on-line only at http://jmb.or.kr.

## References

[1] Mussagy CU, Winterburn J, Santos-Ebinuma VC, Pereira JFB (2019). Production and extraction of carotenoids produced by microorganisms. Appl. Microbiol. Biotechnol..

[2] Sen T, Barrow CJ, Deshmukh SK (2019). Microbial pigments in the food industry-Challenges and the way forward. Front. Nutr..

[3] Garrido-Fernández J, Maldonado-Barragán A, Caballero-Guerrero B, Hornero-Méndez D, Ruiz-Barba JL (2010). Carotenoid production in *Lactobacillus plantarum*. Int. J. Food Microbiol..

[4] Siezen RJ, van Hylckama Vlieg JE (2011). Genomic diversity and versatility of *Lactobacillus plantarum*, a natural metabolic engineer. Cell. Fact..

[5] Zago M, Fornasari ME, Carminati D, Burns P, Suàrez V, Vinderola G (2011). Characterization and probiotic potential of *Lactobacillus plantarum* strains isolated from cheeses. Food Microbiol..

[6] Lee PC, Schmidt-Dannert C (2002). Metabolic engineering towards biotechnological production of carotenoids in microorganisms. Appl. Microbiol. Biotechnol..

[7] Turpin W, Renaud C, Avallone S, Hammoumi A, Guyot J-P, Humblot C (2016). PCR of *crtN*M combined with analytical biochemistry: An efficient way to identify carotenoid producing lactic acid bacteria. Syst. Appl. Microbiol..

[8] Kim M, Seo D-H, Park Y-S, Cha I-T, Seo M-J (2019). Isolation of *Lactobacillus plantarum* subsp. *plantarum* producing C_30_ carotenoid 4,4′-diaponeurosporene and the assessment of its antioxidant activity. J. Microbiol. Biotechnol..

[9] Kim M, Jung D-H, Seo D-H, Chung W-H, Seo M-J (2020). Genome analysis of *Lactobacillus plantarum* subsp. *plantarum* KCCP11226 reveals a well-conserved C_30_ carotenoid biosynthetic pathway. 3 Biotech..

[10] Sachindra NM, Sato E, Maeda H, Hosokawa M, Niwano Y, Kohno M (2007). Radical scavenging and singlet oxygen quenching activity of marine carotenoid fucoxanthin and its metabolites. J. Agric. Food Chem..

[11] Zhang Y, Fang H, Xie Q, Sun J, Liu R, Hong Z (2014). Comparative evaluation of the radical-scavenging activities of fucoxanthin and its stereoisomers. Molecules.

[12] Bergamini CM, Gambetti S, Dondi A, Cervellati C (2004). Oxygen, reactive oxygen species and tissue damage. Curr. Pharm. Design.

[13] Young AJ, Lowe GM (2018). Carotenoids-antioxidant properties. Antioxidants.

[14] Hagi T, Kobayashi M, Kawamoto S, Shima J, Nomura M (2013). Expression of novel carotenoid biosynthesis genes from *Enterococcus gilvus* improves the multistress tolerance of *Lactococcus lactis*. J. Appl. Microbiol..

[15] Steiger S, Perez-Fons L, Fraser P, Sandmann G (2012). Biosynthesis of a novel C_30_ carotenoid in *Bacillus firmus* isolates. J. Appl. Microbiol..

[16] Young AJ, Lowe GM (2001). Antioxidant and prooxidant properties of carotenoids. Arch. Biochem. Biophys..

[17] Ekmekci H, Aslim B, Ozturk S (2009). Characterization of vaginal lactobacilli coaggregation ability with Escherichia coli. Microbiol. Immunol..

[18] Kim SH, Kim MS, Lee BY, Lee PC (2016). Generation of structurally novel short carotenoids and study of their biological activity. Sci. Rep..

[19] Chae HS, Kim K-H, Kim SC, Lee PC (2010). Strain-dependent carotenoid productions in metabolically engineered *Escherichia coli*. Appl. Biochem. Biotechnol..

[20] Wieland B, Feil C, Gloria-Maercker E, Thumm G, Lechner M, Bravo J-M (1994). Genetic and biochemical analyses of the biosynthesis of the yellow carotenoid 4,4′-diaponeurosporene of *Staphylococcus aureus*. J. Bacteriol..

[21] Yang J, Li Y, Zhang L, Fan M, Wei X (2017). Response surface design for accumulation of selenium by different lactic acid bacteria. 3 Biotech..

[22] Han Q, Kong B, Chen Q, Sun F, Zhang H (2017). *In vitro* comparison of probiotic properties of lactic acid bacteria isolated from Harbin dry sausages and selected probiotics. J. Funct. Food.

[23] Tulumoglu S, Yuksekdag ZN, Beyatli Y, Simsek O, Cinar B, Yaşar E (2013). Probiotic properties of lactobacilli species isolated from children's feces. Anaerobe.

[24] García-Cayuela T, Korany AM, Bustos I, de Cadiñanos LPG, Requena T, Peláez C (2014). Adhesion abilities of dairy *Lactobacillus plantarum* strains showing an aggregation phenotype. Food Res..

[25] Hu P-L, Yuan Y-H, Yue T-L, Guo C-F (2018). A new method for the in vitro determination of the bile tolerance of potentially probiotic lactobacilli. Appl. Microbiol. Biotechnol..

[26] Frengova GI, Beshkova DM (2009). Carotenoids from *Rhodotorula* and *Phaffia*: yeasts of biotechnological importance. J. Ind. Microbiol. Biotechnol..

[27] Simpson KL, Nakayama T, Chichester C (1964). Biosynthesis of yeast carotenoids. J. Bacteriol..

[28] Polulyakh OV, Podoprigora OI, Eliseev SA, Ershov YV, Bykhovskii VY, Dmitrovskii AA (1992). Biosynthesis of torulene and torularhodin in the yeast *Phaffia rhodozyma*. Appl. Biochem. Microbiol..

[29] Bhosale P (2004). Environmental and cultural stimulants in the production of carotenoids from microorganisms. Appl. Microbial. Biotechnol..

[30] Roginsky V, Lissi EA (2005). Review of methods to determine chain-breaking antioxidant activity in food. Food Chem..

[31] Méndez-Robles MD, Permady HH, Jaramillo-Flores ME, Lugo-Cervantes EC, Cardador-Martínez A, Canales-Aguirre AA (2006). C-26 and C-30 Apocarotenoids from seeds of Ditaxis heterantha with antioxidant activity and protection against DNA oxidative damage. J. Nat. Prod..

[32] Hinneburg I, Dorman HD, Hiltunen R (2006). Antioxidant activities of extracts from selected culinary herbs and spices. Food Chem..

[33] Müller L, Fröhlich K, Böhm V (2011). Comparative antioxidant activities of carotenoids measured by ferric reducing antioxidant power (FRAP), ABTS bleaching assay (αTEAC), DPPH assay and peroxyl radical scavenging assay. Food Chem..

