# Supplementary material for: 4,4′-Diaponeurosporene from Lactobacillus plantarum subsp. plantarum KCCP11226: Low Temperature Stress-Induced Production Enhancement and In Vitro Antioxidant Activity
Source: J Microbiol Biotechnol. 2020 Nov 4;31(1):63–9. doi: 10.4014/jmb.2010.10022 (PMC9706005; doi:10.4014/jmb.2010.10022)
Supplement: Supplementary file 1 [file jmb-31-1-63-supple.pdf]

J. Microbiol. Biotechnol.
https://doi.org/10.4014/jmb.2010.10022

Table S1. Antibiotic resistance profiles of *Lactobacillus plantarum* subsp. *plantarum* KCCP11226

Strain	Antibiotics							
	Amp[a]	Ery[b]	Gen[c]	Cip[d]	Lin[e]	Nov[f]	Tet[g]	Strep[h]
L. plantarum subsp. *plantarum* KCCP11226	S	R	R	R	R	S	R	R

S, sensitive; R, resistant

[a]Ampicillin; [b]Erythromycin; [c]Gentamicin; [d]Ciprofloxacin; [e]Lincomycin; [f]Novobiocin; [g]Tetracycline; [h]Streptomycin

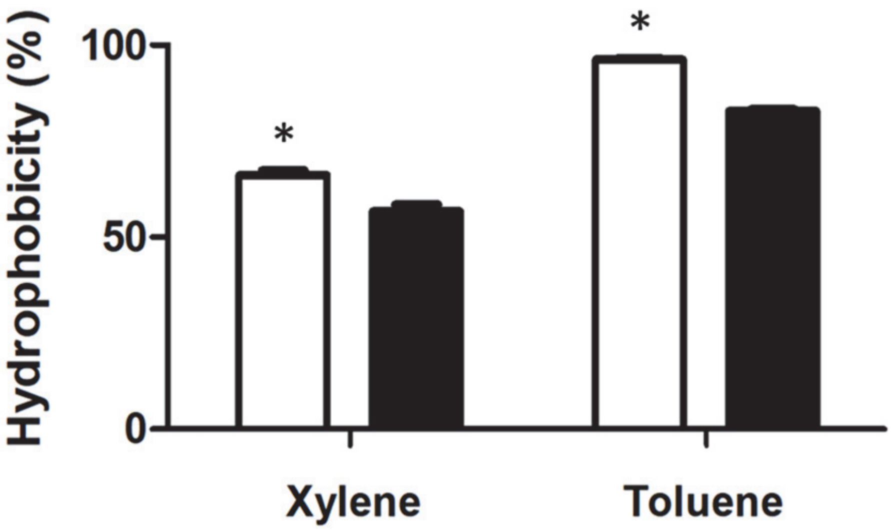

Fig. S1. Hydrophobicity against xylene and toluene. White and black bars indicate *L. plantarum* subsp. *plantarum* KCCP11226 and *L. rhamnosus* GG, respectively. The results from three independent test are represent as means ± SD. Significant means were expressed after analysis of variance (ANOVA) analysis with Tukey-Kramer multiple comparison tests (*$p < 0.01$).
